# Recent advances in electrochemical copper catalysis for modern organic synthesis

**DOI:** 10.3762/bjoc.21.9

**Published:** 2025-01-16

**Authors:** Yemin Kim, Won Jun Jang

**Affiliations:** 1 Department of Chemistry and Nanoscience, Ewha Womans University, Seoul, 03760, Korea

**Keywords:** copper, electrochemistry, radical chemistry, single-electron transfer, sustainable catalysis

## Abstract

In recent decades, organic electrosynthesis has emerged as a practical, sustainable, and efficient approach that facilitates valuable transformations in synthetic chemistry. Combining electrochemistry with transition-metal catalysis is a promising and rapidly growing methodology for effectively forming challenging C–C and C–heteroatom bonds in complex molecules in a sustainable manner. In this review, we summarize the recent advances in the combination of electrochemistry and copper catalysis for various organic transformations.

## Introduction

Transition-metal-catalyzed cross-coupling has emerged as an effective method for forming carbon–carbon (C–C) and carbon–heteroatom (C–X, where X = N, O, or halogens) bonds in organic synthesis. Copper was one of the first transition metals employed in cross-coupling to form C–C and C–X bonds [1–2]. In 1901, Ullmann reported the first cross-coupling reaction for the formation of biaryl compounds in the presence of stoichiometric quantities of a copper reagent [3]. This pioneering work, known as the “classical Ullmann reaction”, was extended by Ullmann and Goldberg to enable the C–N and C–O bond formation [4–6]. Subsequently, key developments in Cu-catalyzed cross-coupling reactions were achieved, including the Rosenmund–von Braun reaction [7], Hurtley’s coupling [8], and the Cadiot–Chodkiewicz reaction [9]. However, these classical reactions often restrict the substrate scope and functional group compatibility due to the harsh conditions required, such as strong bases, high temperatures, and stoichiometric amounts of copper reagents. Consequently, investigation into more practical and sustainable reactions remains an area of ongoing research [10].

**Figure 1:**
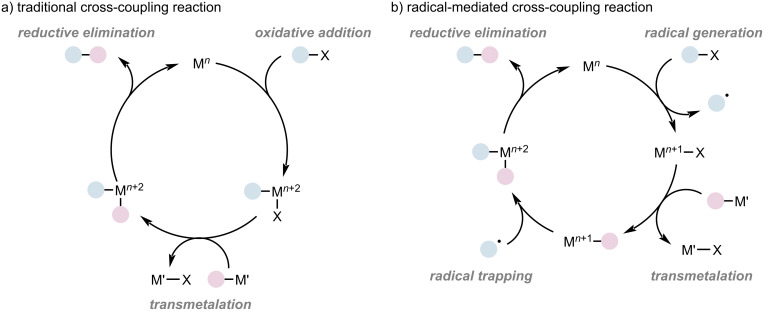
General mechanisms of traditional and radical-mediated cross-coupling reactions.

Conventional cross-coupling reactions typically require C(sp^2^)-based electrophiles and nucleophiles as coupling partners. Generally, the reaction is initiated through oxidative addition, followed by transmetalation and reductive elimination, to obtain the desired product. Throughout the catalytic cycle, the catalyst undergoes conversion between [M]*^n^* and [M]*^n^*^+2^ (Figure 1) [11]. However, using alkyl electrophiles as coupling partners in cross-coupling reactions remains a significant challenge owing to the high energy barrier required for oxidative addition and facile β-hydride elimination [12]. The development of radical approaches facilitated by transition-metal catalysis has provided a promising solution to overcome the limitations of conventional coupling reactions, particularly in controlling the high reactivity and selectivity of radical intermediates [13–14]. Early studies on copper-mediated radical reactions, such as Julia’s work on radical cyclization reaction [15], along with advancements in dimerization [16–17], oxidative cleavage [18–19], and olefin addition reactions [20] conducted by various research groups, contributed to this area of research. Recently, the coupling reactions of C(sp^3^)-based electrophiles were explored using dual photoredox and copper catalysis, achieving selective radical coupling reactions involving alkyl halides [21–24]. Moreover, copper-catalyzed asymmetric radical cross-coupling has advanced significantly over the past decade [25–27], with notable examples including Liu and Stahl’s enantioselective cyanation of benzylic C–H bonds using a Cu/chiral bisoxazoline catalyst [28], along with the Peters’ and Fu’s asymmetric C–N bond cross-coupling reactions by merging photoredox catalysis with copper catalysis [29–30].

**Figure 2:**
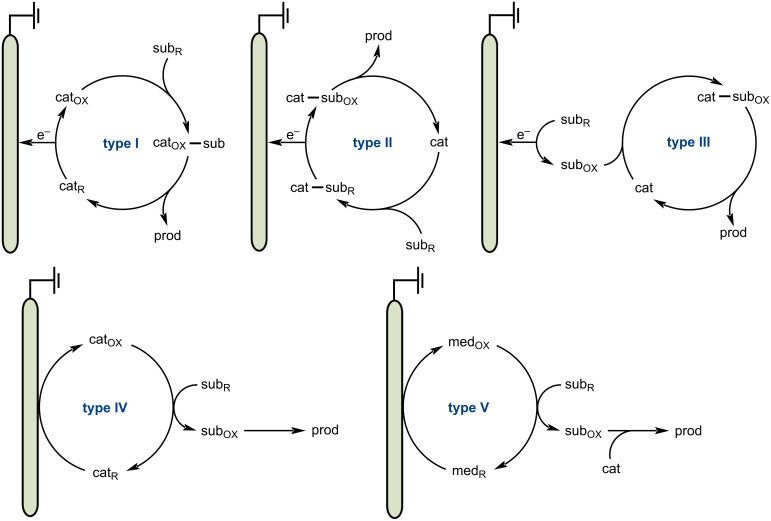
Types of electrocatalysis (using anodic oxidation).

Building on the success of photoredox catalysis, electrochemistry has emerged as a complementary and attractive strategy for promoting sustainability of organic synthesis. By offering viable alternatives to conventional chemical oxidizing and reducing agents [31], electrochemical reactions not only enable substrates to undergo single-electron transfer at the cathode or anode, either directly or indirectly, generating highly reactive radical intermediates, but also allow direct electron transfer to the metal catalyst without the need for chemical redox agents, thus providing milder and more sustainable reaction conditions (Figure 2) [32]. Electrochemical reactions can be performed at low potentials, thereby suppressing side reactions, and chemoselectivity and reactivity can be achieved by precisely controlling the potential. Additionally, the merging of electrochemistry and transition-metal catalysis offers advantages in controlling substrate activation, intermediate reactivity, and bond formation, as well as facilitating asymmetric transformations. As a result, electrochemical reactions have become valuable tools in modern synthetic chemistry. Over the past 15 years (since ca*.* 2010), synthetic organic electrochemistry has undergone remarkable growth, enabling the development of new types of reactions [33].

Numerous review articles have been published [32–38], however, no comprehensive review focusing on Cu-catalyzed electrochemistry has been reported to date. Copper catalysts are potential candidates for pharmaceutical applications owing to their abundance, low cost, and lower toxicity compared with noble transition metals such as palladium [39]. In terms of sustainable chemistry, the combination of copper catalysis and electrochemistry is particularly attractive for overcoming challenges associated with conventional methods, and it has led to extensive research in recent years.

**Figure 3:**
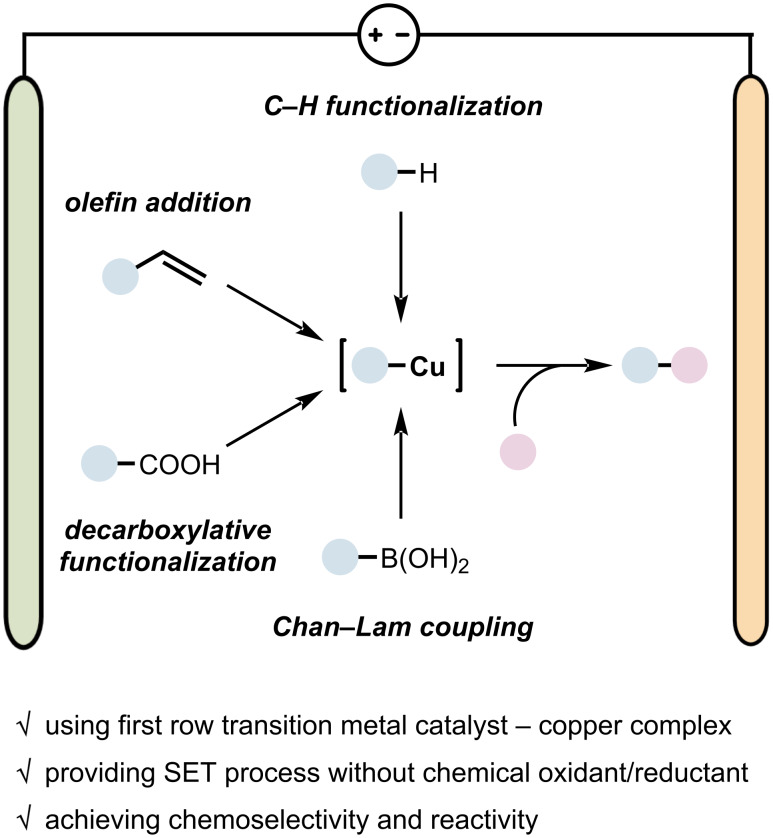
Recent developments and features of electrochemical copper catalysis.

In this review, we highlight the unique contributions of electrochemical copper catalysis to organic synthesis, focusing on recent developments in Cu-catalyzed electrochemical reaction categorized into four types: 1) C–H functionalization, 2) olefin addition, 3) decarboxylative functionalization, and 4) coupling reactions (Figure 3). This review aims to provide insight into the potential of copper-based electrochemical methodologies while also inspiring future research in this rapidly growing field.

## Review

### C–H Functionalization

Site- and chemoselective C–H functionalization has emerged as a powerful platform for the formation of new C–C and C–heteroatom bonds, offering an efficient and economical approach for molecular synthesis [40]. This strategy has been widely applied in synthetic chemistry, the pharmaceutical industry, and materials science. Over the past few decades, transition-metal-catalyzed C–H activation reactions have been widely developed. Late-stage C–H functionalization of highly complex and diverse molecules, such as those of pharmaceuticals and natural products, has provided new retrosynthetic disconnections for complex compounds, contributing to improved resource efficiency [41–46]. Recently, the merging of C–H activation and electrochemistry has emerged as a potential synthetic tool for the formation of new C–C, and C–heteroatom bonds using electricity to replace the stoichiometric amounts of conventional chemical redox reagents [47].

#### C–C Bond formation

**Figure 4:**
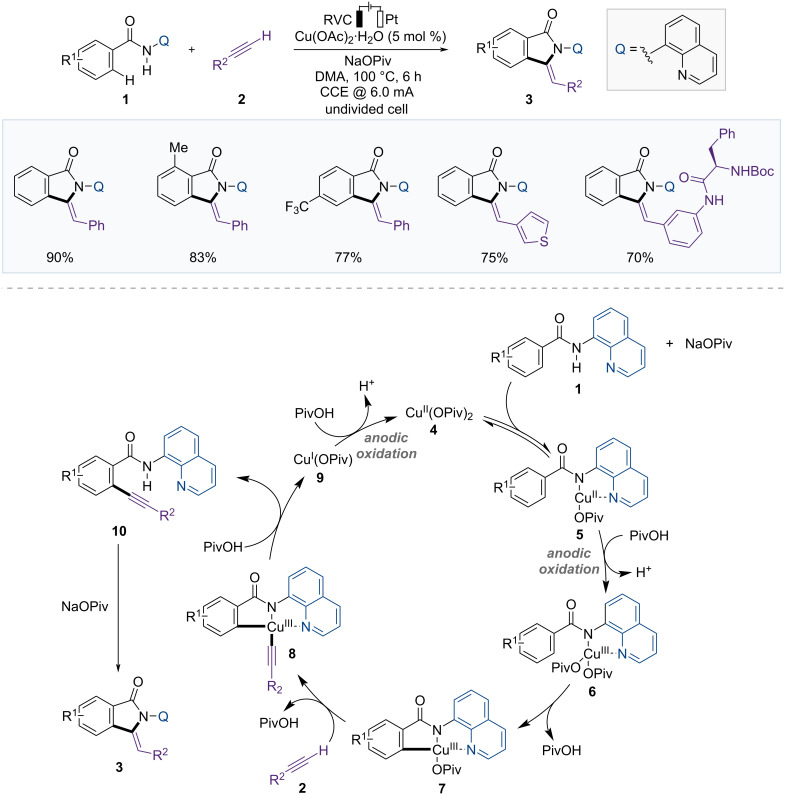
Scheme and proposed mechanism for Cu-catalyzed alkynylation and annulation of benzamide.

In 2019, the Ackermann group established a synthetic method for isoindolones using a Cu-catalyzed electrochemical C–H activation strategy through C–H alkynylation of arylamides followed by electrooxidative cascade annulation (Figure 4) [48]. This reaction enables sustainable C–H functionalization by utilizing electricity as the terminal oxidant instead of stoichiometric amounts of toxic chemical oxidants and releasing hydrogen gas as the sole byproduct. Various benzamides **1** and terminal arylalkynes **2** bearing electron-rich or electron-withdrawing groups provided the desired products **3** with high chemoselectivities. However, terminal alkynes with alkyl substituents did not yield the desired annulation products. Moreover, the same products were generated using alkynyl carboxylic acids instead of terminal alkynes via decarboxylative C–H alkynylation and annulation.

Cyclic voltammetry (CV) studies exhibited an oxidative current at 0.95 V vs SCE in the presence of the Cu(II) salt, base, and benzamide, however, no relevant competitive oxidation peak was observed with only Cu(OAc)_2_. These results indicate that Cu(II) intermediate **5** was generated. Based on the mechanistic studies, the authors suggested plausible reaction mechanisms (Figure 4). First, the Cu(II) catalyst coordinates with substrate **1** in the presence of a base to form Cu(II) complex **5**, which undergoes anodic oxidation to generate Cu(III) intermediate **6**. Carboxylate-assisted C–H activation of the benzamide subsequently leads to the formation of Cu(III) species **7**. Metalation of the terminal alkyne **2**, followed by reductive elimination, produces C–H alkynylated arene **10**, which then forms the final product **3** through intramolecular cyclization. Finally, the Cu(I) complex **9** produced via reductive elimination is reoxidized at the anode to regenerate the Cu(II) complex **4**, completing the catalytic cycle.

**Figure 5:**
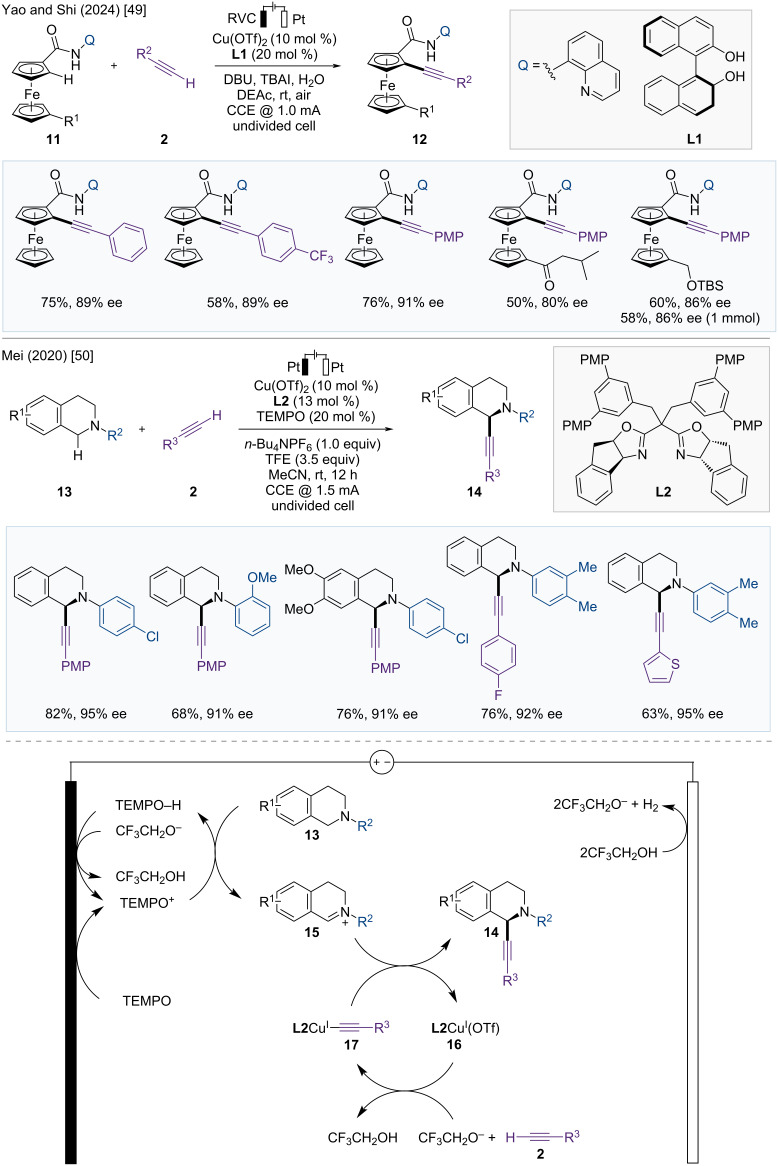
Scheme and proposed mechanism for Cu-catalyzed asymmetric C–H alkynylation.

Yao and Shi developed the enantioselective C–H alkynylation of ferrocene carboxamides with terminal alkynes by using Cu/BINOL and an electrocatalytic system (Figure 5) [49]. 8-Aminoquinoline-assisted C–H functionalization provided planar chiral ferrocenes with high yield and enantioselectivity. This reaction can be applied to a wide range of substrates, including arylacetylenes with electron-donating and electron-withdrawing groups, and ferrocenyl amides with alkyl and acyl substituents on the other Cp ring. Additionally, the reaction showed similar reactivity and enantioselectivity on a 1 mmol scale.

In 2020, Mei et al. reported the asymmetric C(sp^3^)–H alkynylation of tertiary cyclic amines by merging Cu(II)/TEMPO catalysis with electrochemistry to yield chiral C1-alkynylated tetrahydroisoquinolines (THIQs) (Figure 5) [50]. As a co-catalytic redox mediator, TEMPO plays an essential role in the formation of iminium intermediate **15** and in decreasing the oxidation potential. A range of functional groups, such as halides, ethers, and heterocycles, were tolerated well, yielding the corresponding enantioenriched products **14** with high enantioselectivity in the presence of chiral bisoxazoline ligand **L2**.

A possible mechanism is depicted in Figure 5. First, TEMPO is converted to TEMPO^+^ through anodic oxidation, and iminium intermediate **15** is created through hydride transfer from THIQ (**13**) to TEMPO^+^. TEMPO–H, generated during the hydrogen transfer step, then returns to TEMPO^+^ through anodic oxidation. Chiral acetylide species **17** is produced from the terminal alkyne **2** in the presence of a chiral copper catalyst and base, which reacts with the electrophilic iminium intermediate **15** to yield the desired chiral product **14**. Active Cu(I) is regenerated either through cathodic reduction or by reaction with TEMPO–H.

A year after the Mei group’s report, the Xu group developed the electrocatalytic racemic C(sp³)–H alkynylation of THIQs with terminal alkynes in a continuous-flow microreactor using copper/TEMPO relay catalysis [51]. The electrocatalytic reaction in continuous flow facilitates straightforward scale-up and demonstrating a broad substrate scope.

**Figure 6:**
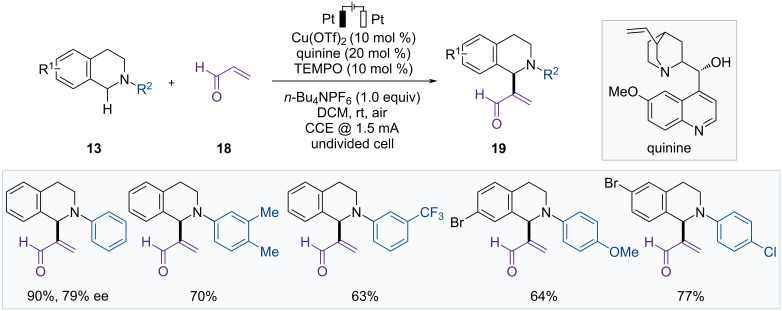
Scheme for Cu/TEMPO-catalyzed C–H alkenylation of THIQs.

In 2023, the Mei group reported the C(sp^3^)–H alkenylation of THIQs with acrolein by a combination of Cu/TEMPO and electrooxidation (Figure 6) [52]. CV experiments demonstrated that TEMPO was a suitable redox mediator, and on/off experiments confirmed that the reaction continued even without electrolysis. THIQ (**13**) was rapidly oxidized to an iminium intermediate under the action of electricity and oxygen. However, when the iminium intermediate was converted to the desired product **19**, electrolysis had no effect, and this step proceeded more gradually than the initial oxidation step. Therefore, the optimized conditions allowed the reaction to proceed under a constant current electrolysis at 1.5 mA for 6 hours, followed by stirring for additional 24 hours with the electricity turned off. These reaction conditions were applicable to various *N*-aryl-THIQ derivatives with various functional groups. Using quinine as a chiral ligand under standard conditions, the chiral product was obtained with a high yield and 79% ee.

**Figure 7:**
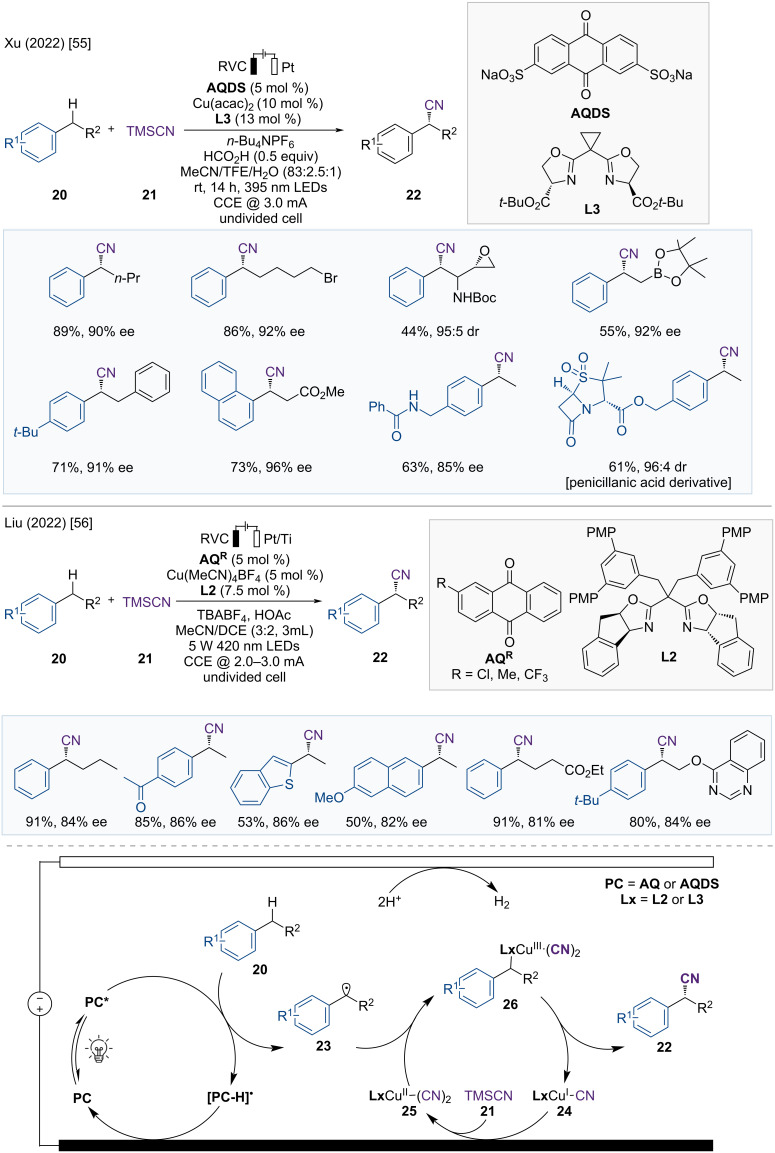
Scheme and proposed mechanism for Cu-catalyzed electrophotochemical enantioselective cyanation of benzylic C(sp³)–H bonds.

Enantioselective C(sp^3^)–H functionalization is an attractive strategy for synthesizing chiral molecules. Significant progress has been achieved in transition-metal-catalyzed asymmetric C–H functionalization with directing groups, however, achieving similar results without the assistance of a directing group continues to be a significant challenge [53–54]. Radical-based approaches can facilitate C(sp³)–H functionalization without directing groups, however, controlling the selectivity is difficult. In 2022, Xu and co-workers established a site- and enantioselective cyanation of benzylic C(sp³)–H bonds using an electro-photochemical strategy (Figure 7) [55]. The reaction conditions show a broad substrate tolerance, and the late-stage functionalization of complex molecules derived from natural products and drugs has proven to be useful in these reactions.

As shown in Figure 7, the photocatalyst sodium anthraquinone-2,7-disulfonate (**AQDS**) is excited by 395 nm light to form AQDS* and undergoes electron transfer with arylalkanes **20** to generate an ion-radical pair (**AQDS^•−^**, **20^•+^**). This ion radical pair (**AQDS**^•^**^−^**, **20**^•^**^+^**) then generate a benzylic radical **23** and a semiquinone radical (**[AQDS–H]^•^**) through proton transfer. The benzylic radical intermediate **23** subsequently reacts with the chiral copper catalyst **L3**Cu(II)(CN)_2_ (**25**) to form a Cu(III) complex **26**, which undergoes reductive elimination to produce a chiral product **22**. The reduced copper catalyst **24** and **[AQDS–H]^•^** are reoxidized to **L3**Cu(II)(CN)_2_ (**25**) and **AQDS** at the anode, respectively, completing the catalytic cycle.

In the same year, following a similar approach, the Liu group explored a Cu-catalyzed photoelectrochemical enantioselective cyanation of benzylic C(sp^3^)–H bonds (Figure 7) [56]. A wide range of electron-poor and electron-rich alkylarenes **20** are suitable substrates for this electrophotocatalytic radical relay strategy. Additionally, late-stage functionalization of bioactive molecules provides the corresponding chiral cyanation products with high enantioselectivity.

The catalytic cycle is depicted in Figure 7. The photoexcited photocatalyst anthraquinone (AQ*) acts as a hydrogen-atom transfer (HAT) acceptor and transforms the alkylarene **20** into benzylic radical intermediate **23** together with reduced **[AQ–H]**^•^. The benzylic radical intermediate **23** is captured by the **L2**Cu(II)(CN)_2_ complex **25** and then undergoes reductive elimination to provide the chiral nitrile product **22**. Finally, the reduced **[AQ–H]**^•^ and **L2**Cu(I)CN (**24**) are reoxidized at the anode to complete the catalytic cycle.

**Figure 8:**
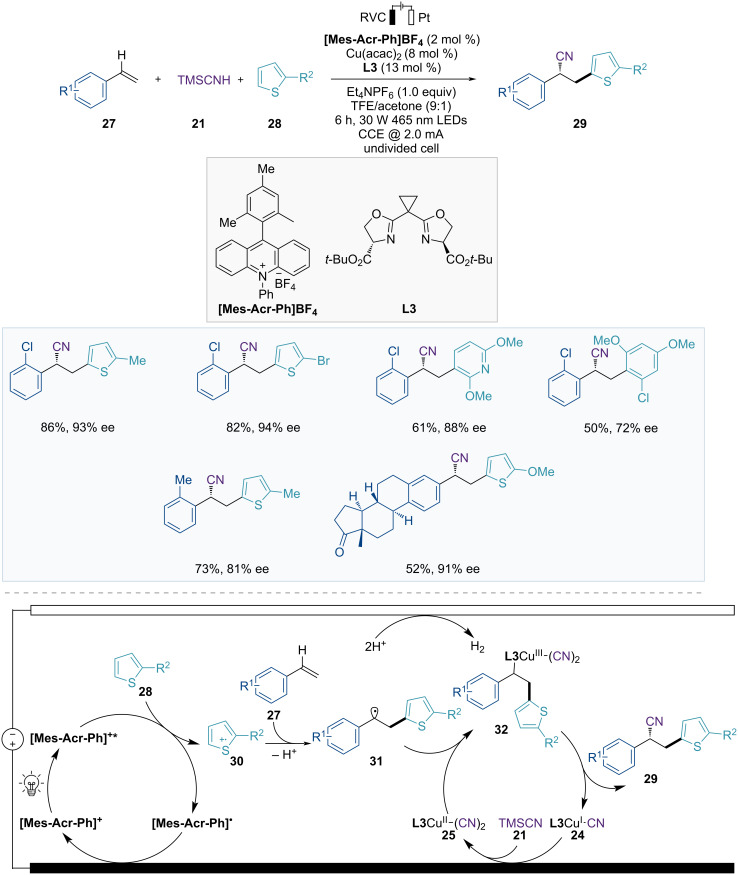
Scheme and proposed mechanism for Cu-catalyzed electrophotochemical asymmetric heteroarylcyanation of alkenes.

In 2023, Xu and Lai developed a three-component system for the enantioselective dicarbofunctionalization of olefins, using photoelectrocatalysis with asymmetric copper catalysis (Figure 8) [57]. This asymmetric heteroarylcyanation of arylalkenes **27** via C–H functionalization has a broad substrate scope, including various arylalkenes and heteroarenes, yielding enantioenriched nitrile products **29**.

The proposed mechanism is illustrated in Figure 8. [Mes-Acr-Ph]^+^* is generated through the photoexcitation of the photocatalyst **[Mes-Acr-Ph]^+^**, which undergoes electron transfer to the heteroarene **28**, resulting in the formation of the **[Mes-Acr-Ph]^•^** and heteroarene radical cation **30**. The **[Mes-Acr-Ph]^•^** is regenerated to the ground-state acridinium **[Mes-Acr-Ph]^+^** through a single oxidation step on the anode, and the heteroarene radical cation **30** then reacts with the arylalkene **27** to form a benzylic radical intermediate **31**. The benzylic radical intermediate **31** is subsequently captured by a chiral Cu(II) complex **25** to generate the Cu(III) complex **32**. Subsequent reductive elimination provides the chiral product **29** and the Cu(I) complex **24**. The catalytic cycle is completed when the Cu(I) complex **24** is reoxidized to the Cu(II) complex **25** through anodic oxidation.

**Figure 9:**
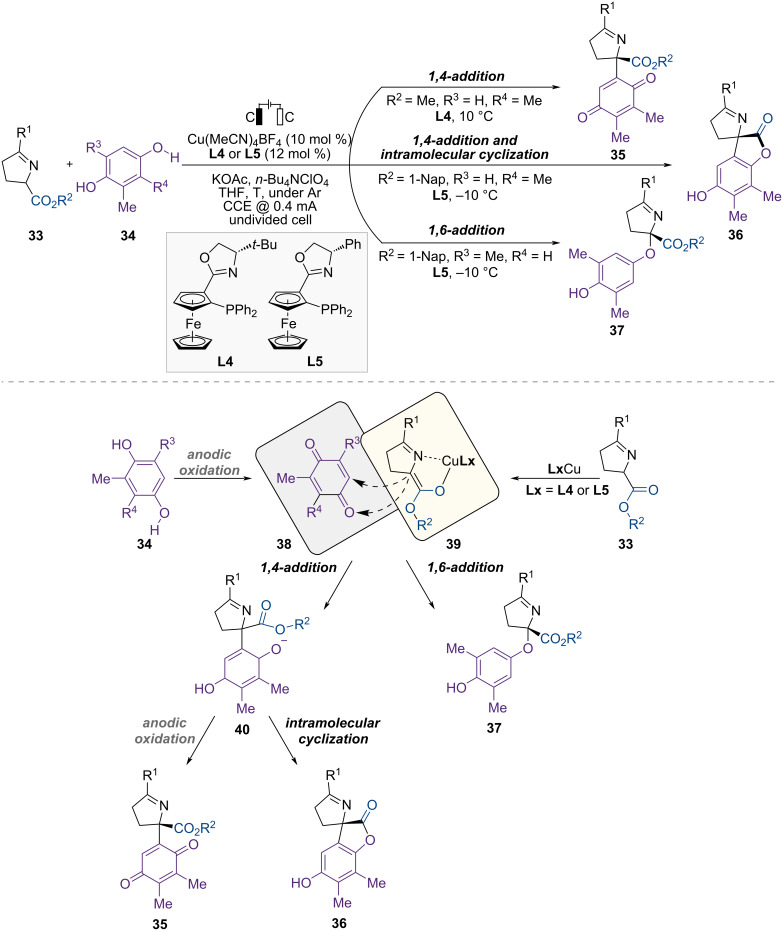
Scheme and proposed mechanism for Cu-catalyzed enantioselective regiodivergent cross-dehydrogenative coupling of Schiff bases with hydroquinones.

In 2023, Guo and co-workers reported Cu-catalyzed asymmetric electrochemical regiodivergent cross-dehydrogenative coupling of Schiff bases and hydroquinones (Figure 9) [58]. In this approach, a chiral copper complex was used as a Lewis acid catalyst, yielding various synthetic routes for synthesizing chiral amino esters containing a quaternary stereocenter, and the control of regioselectivity depended on the bulkiness of the substrates. Additionally, the electrochemical system served as an internal syringe pump, generating quinone from hydroquinone in situ through anodic oxidation, which enhanced the enantioselectivity. First, the reaction of ketimine ester **33** and 2,3-dimethylhydroquinone at 10 °C provided the chiral 1,4-addition product **35** via dynamic kinetic asymmetric transformation (DyKAT). Conversely, when the reaction was performed at −10 °C, the reaction pathway switched from DyKAT to kinetic resolution (KR) of the racemic ketimine ester, providing the same chiral product **35** with recovered enantioenriched starting material. Additionally, when a 1-naphthyl ester was used instead of a methyl ester at −10 °C, 1,4-addition followed by intramolecular tandem annulation generated the corresponding chiral product **36**. Finally, using 1-naphthyl ester and relatively bulkier 2,6-dimethylhydroqunone as starting materials produced chiral 1,6-addition products **37**.

In mechanistic studies, using quinone **38** instead of hydroquinone **34** in the electrochemical-free process produced the desired product **36**, with a similar yield but a significantly lower ee (48%) than that obtained under standard electrochemical conditions. By contrast, when quinone was added gradually over 4.5 h using a syringe pump, the desired product **36** was obtained with a similar yield and enantioselectivity. This result corresponded to that of a standard Cu-catalyzed electrochemical protocol. Based on mechanistic studies, the proposed mechanism is shown in Figure 9. First, hydroquinone **34** is oxidized at the anode to generate a quinone intermediate **38**. Meanwhile, the chiral copper catalyst reacts with the Schiff base **33**, generating a nucleophilic copper-coordinated azomethine ylide **39**. Subsequently, the chiral products **35**–**37** are produced through the reaction between the metalated azomethine ylide **39** and the quinone intermediate **38**. The reaction pathway, either 1,4-addition or 1,6-addition, depends on the structure of the hydroquinone **34**. The less sterically hindered hydroquinone promotes 1,4-addition, resulting in the formation of an α-arylated intermediate **40**, and different products are generated depending on the substituents on the Schiff base. For example, a methyl-substituted Schiff base provided a chiral quinone **35** after 1,4-addition and electrochemical oxidation. In contrast, the naphthyl-substituted Schiff base generated the corresponding enantioenriched product **36** through 1,4-addition followed by intramolecular annulation. When 2,6-disubstituted hydroquinone was used as the starting material, 1,6-addition occurred due to steric hindrance, yielding an α-aryloxylation product **37**.

**Figure 10:**
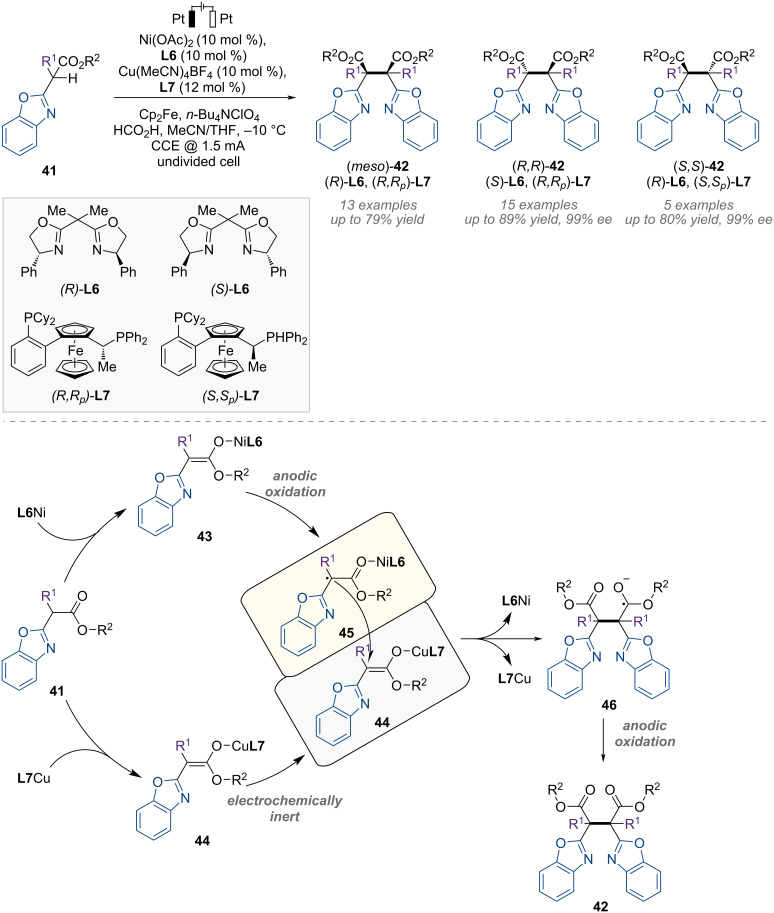
Scheme and proposed mechanism for Cu/Ni-catalyzed stereodivergent homocoupling of benzoxazolyl acetate.

After their investigation of Cu-catalyzed electrochemical reactions, the same group further developed synergistic Cu/Ni catalysis for the stereodivergent electrooxidation of benzoxazolyl acetate (Figure 10) [59].

In this catalytic system, copper and nickel activate identical racemic carbonyl nucleophiles to generate Cu-enolate **44** and Ni-enolate **43** simultaneously (Figure 10). The Ni-enolate **43** undergoes anodic oxidation through single-electron transfer, releasing nickel-bound α-carbonyl radical **45**, whereas the copper complex **44** remains electrochemically inert under standard conditions. Subsequently, radical-polar coupling between electrophilic Ni-bound α-carbonyl radical intermediate **45** and remaining nucleophilic Cu-enolate **44** provides a chiral product **42** containing vicinal quaternary stereocenters with high stereoselectivity, and all three possible stereoisomers of the product are accessible by adjusting the two distinct chiral catalysts.

#### C–N Bond formation

**Figure 11:**
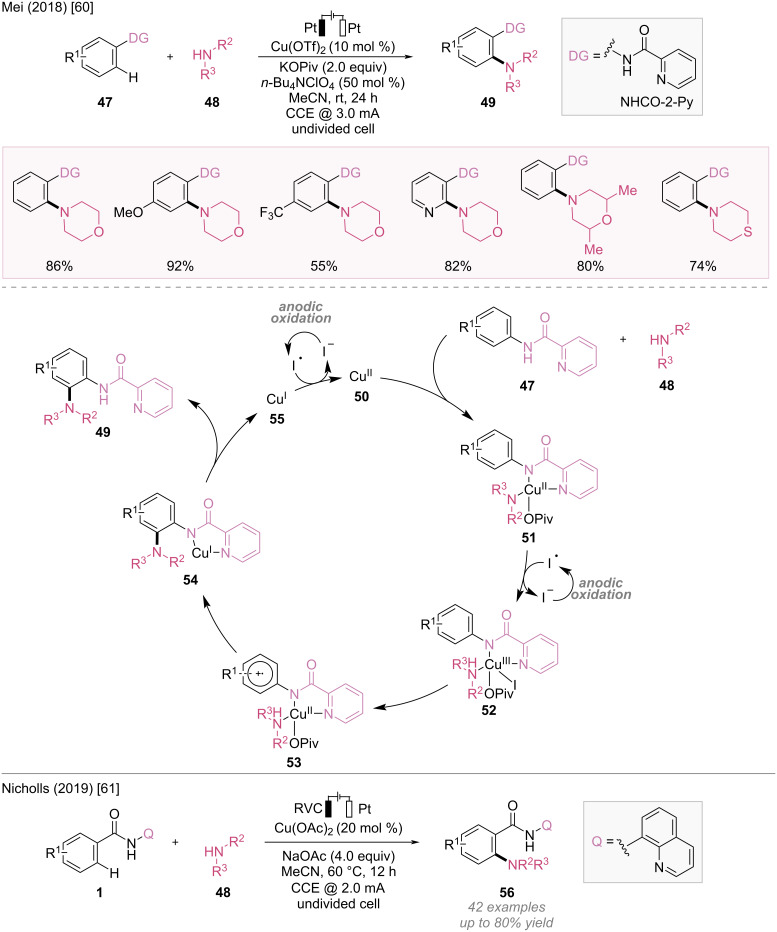
Scheme and proposed mechanism for Cu-catalyzed electrochemical amination.

In 2018, Mei et al. developed the electrochemical C–H amination of arenes with amine electrophiles using copper catalysis, which provided a step-economical approach for the synthesis of aromatic amines by employing electricity as an oxidant (Figure 11) [60].

Mechanistic studies have indicated that *n*-Bu_4_NI acts as a redox mediator at the anode, and the electron transfer between the copper complex and the iodine radical is the rate-determining step. The author proposed a catalytic cycle, as illustrated in Figure 11. Initially, the Cu(II) catalyst **50** coordinates with substrate **47** and amine electrophile **48** to generate Cu(II) intermediate **51**, which is then oxidized by the iodine radical to form Cu(III) complex **52**. Cu(III) complex **52** undergoes electron transfer to produce radical cation intermediate **53**. Subsequent intramolecular amine transfer to the radical cation intermediate **53**, followed by ligand exchange, yields amination product **49** and Cu(I) species **55**. Cu(II) catalyst **50** is regenerated by anodic oxidation, thereby completing the catalytic cycle.

In 2019, Nicholls et al. reported a Cu-catalyzed directed C–H amination of benzamides with secondary amine electrophiles independently (Figure 11) [61].

**Figure 12:**
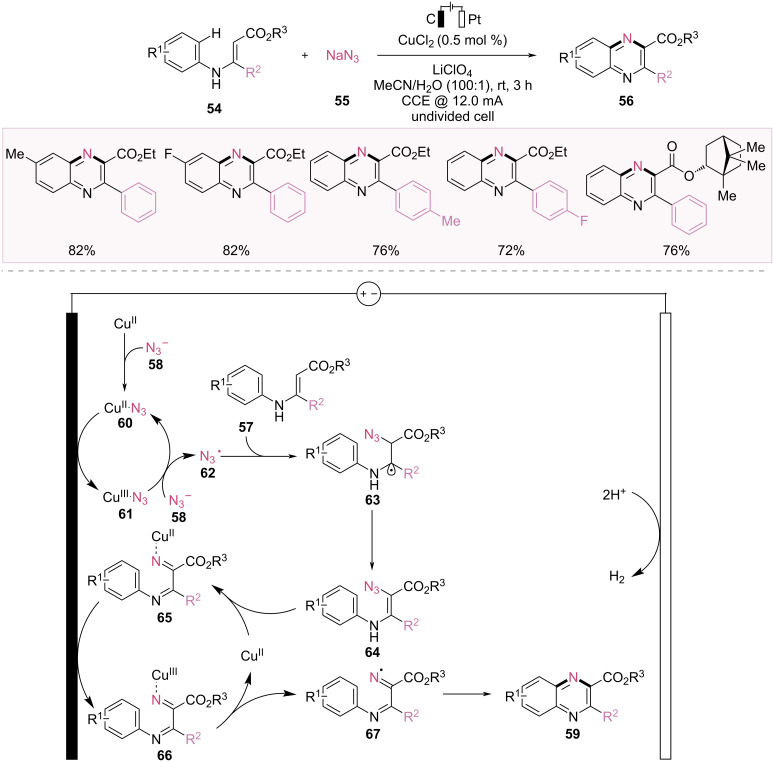
Scheme and proposed mechanism for Cu-catalyzed electrochemical azidation of *N*-arylenamines and annulation.

In 2023, De Sarkar and Baidya reported the Cu-catalyzed electrocatalytic azidation of N-arylenamines, followed by denitrogenative annulation for quinoxaline synthesis (Figure 12) [62]. Only 0.5 mol % CuCl_2_ catalyst was required, and anodic oxidation was employed instead of stoichiometric chemical oxidants. This cascade strategy is compatible with various substituted *N*-arylenamines **57** that bear electron-withdrawing and electron-donating groups, facilitating the production of quinoxaline scaffolds **59**.

According to the reaction mechanism outlined in Figure 12, the copper catalyst reacts with an azide ion to generate a Cu(II)–N_3_ complex **60**, which is then anodically oxidized to the Cu(III)–N_3_ complex **61**. The Cu(III)–N_3_ complex **61** releases the azidyl radical **62** from the azide ion **58**, returning it to the Cu(II)–N_3_ complex **60**. The azidyl radical **62** then reacts with *N*-arylenamine **57** via radical addition. Thereafter, it undergoes oxidation to form a kinetically labile vinyl azide intermediate **64**. This vinyl azide intermediate **64** dissociates, yielding Cu(II) iminyl complex **65** via denitrogenation. Accordingly, the formation of the Cu(II) iminyl complex **65** promotes the electrochemical oxidation of Cu(III) iminyl complex **66**, which then dissociates to generate the iminyl radical **67**. Finally, the iminyl radical delivers the quinoxaline product **59** via radical annulation, followed by rearomatization through oxidation.

#### C–X Bond formation

**Figure 13:**
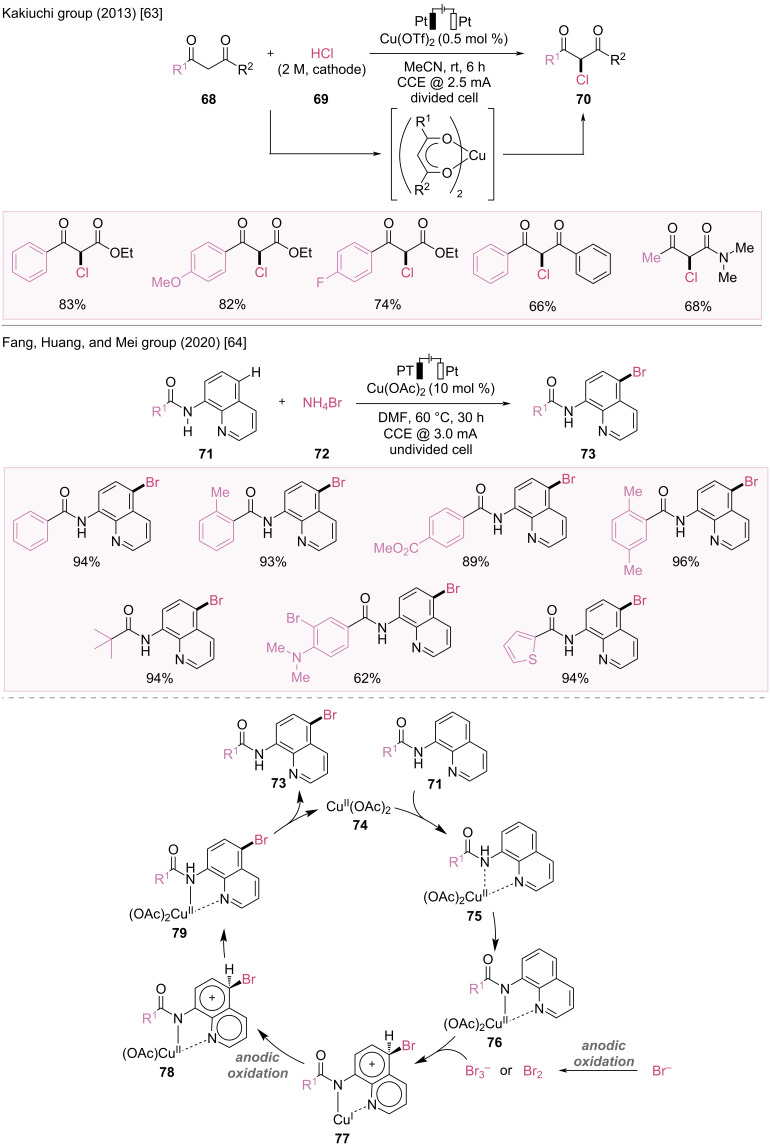
Scheme and proposed mechanism for Cu-catalyzed electrochemical halogenation.

Cu-catalyzed electrochemical reactions have been developed for the formation of C–C, C–N, and C–X bonds. For instance, in 2013, the Kakiuchi group reported Cu-catalyzed electrochemical chlorination of 1,3-dicarbonyl compounds (Figure 13) [63]. Typical chlorination reactions are performed using electrophilic chlorinating reagents or stable and readily available chloride sources with stoichiometric amounts of chemical oxidants. However, in this catalytic system, chloride (Cl^−^) from HCl was used as the chlorinating agent, and electrophilic chlorine (Cl^+^) was generated in situ by the anodic oxidation of chloride ions, thus replacing stoichiometric chemical oxidants. This catalytic electrochemical chlorination method is suitable for β-ketoesters **68** with electron-withdrawing or electron-donating groups on aryl substituents, as well as for β-diketone, and β-ketoamides.

In 2020, Fang, Huang, and Mei et al. explored Cu-catalyzed electrochemical C(sp^2^)–H bromination of 8-aminoquinoline amide at the C5 site of quinoline using NH_4_Br as a brominating reagent under anoxic oxidation conditions (Figure 13) [64]. This catalytic reaction has a broad substrate scope, and further investigation of analogous substrates demonstrates that a bidentate nitrogen structure and a free N–H group are essential for this transformation.

The catalytic cycle begins with the coordination of 8-aminoquinoline **71** to Cu(II) catalyst **74**, providing an arylcopper complex **76** (Figure 13). This is followed by a bromine radical attack that leads to the formation of a cationic brominated copper complex **77**. Anodic oxidation and subsequent proton transfer provide the desired product **73** and regenerate the copper catalyst.

### Olefin addition

Hydrofunctionalization and difunctionalization of alkenes are valuable methods for synthesizing complex molecules from alkenes, a readily available feedstock [65]. Particularly, transition-metal-catalyzed difunctionalization has recently been extensively investigated, and asymmetric reactions have been developed [66]. Many approaches rely on the addition of a radical species to an alkene to generate a radical intermediate, followed by oxidation, which enables radical-polar crossover (RPC) and the subsequent nucleophilic attack of the cationic intermediate [67]. Alternatively, the initial radical intermediate can be trapped by a transition-metal catalyst, followed by a cross-coupling approach to generate difunctionalization products through reductive elimination. Recently, electrocatalytic difunctionalization has been developed, and dual catalytic systems combining transition-metal catalysis with electrocatalysis have emerged [68].

**Figure 14:**
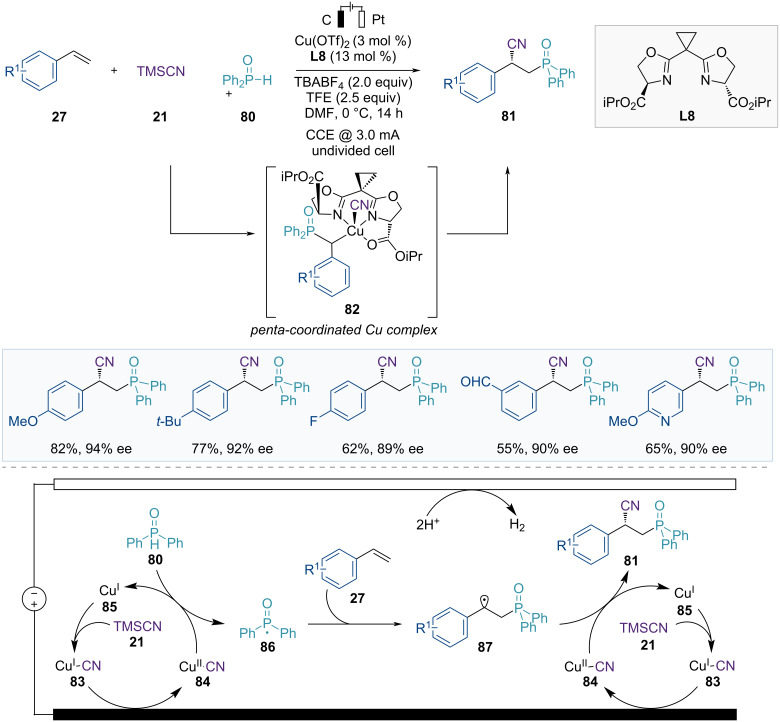
Scheme and proposed mechanism for Cu-catalyzed asymmetric cyanophosphinoylation of vinylarenes.

In 2019, Lin et al. reported the Cu-catalyzed asymmetric electrocatalytic cyanophosphinoylation of vinylarenes (Figure 14) [69]. In the presence of a copper catalyst and the chiral ligand sBOX(iPr) (**L8**) in an electrochemical cell, these three component reactions using styrene derivatives **27**, TMSCN (**21**), and diarylphosphine oxide **80** as starting materials yielded the enantioenriched phosphinoylcyanation products **81** in good yields with high enantioselectivities. Additionally, an appropriate electrolyte (TBABF_4_) and proton source (TFE) were used for this transformation without any conventional chemical oxidants. A key factor in achieving high enantioselectivity is the introduction of serine-derived bisoxazoline ligands **L8** (sBOX). Upon coordination with a copper catalyst, these ligands present second-sphere ester groups, which facilitate the additional stabilization of noncovalent interactions at the penta-coordinated Cu(III) intermediate **82** in the enantio-determining transition states.

Based on the mechanistic studies, a reaction mechanism is proposed in Figure 14. First, the in situ-generated Cu(I)–CN complex **83** is oxidized at the anode to form a Cu(II)–CN complex **84**, which reacts with diarylphosphine oxide **80** to generate a transient P-centered radical **86**. The resulting Cu(I) catalyst **85** is reoxidized at the anode to regenerate the Cu(II)–CN catalyst **84**. The P-centered radical **86** is trapped by the olefin **27** to produce a benzylic radical intermediate **87** that can react with the Cu(II)–CN complex **84** to form an alkyl–Cu(III)–CN intermediate **82**. This intermediate **82** undergoes enantiodetermining reductive elimination to deliver the chiral phosphinoylcyanation products **81** and regenerate the Cu(I) species.

**Figure 15:**
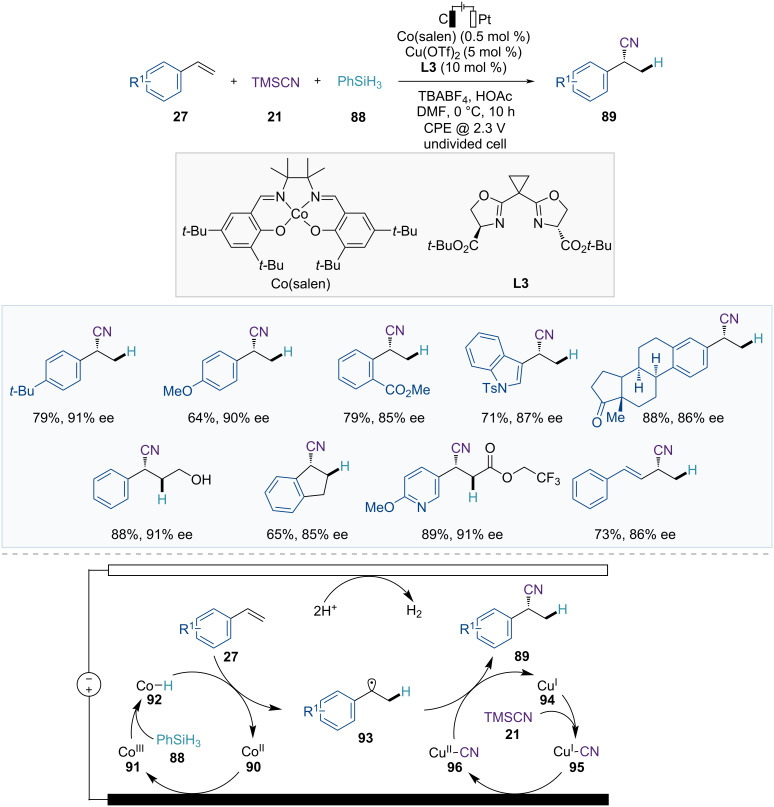
Scheme and proposed mechanism for Cu/Co dual-catalyzed asymmetric hydrocyanation of alkenes.

The Lin group developed an electrochemical approach for the asymmetric hydrocyanation of olefins, facilitated by a Cu/Co dual electrocatalytic system (Figure 15) [70]. In this catalytic system, catalytic amounts of Cu(sBOX) (**L3**) and Co(salen) complexes promote the formation of chiral nitriles **89** in the presence of PhSiH_3_ (**88**) as the hydride source and TMSCN (**21**) as the cyanide source via the effective sequential addition of a hydrogen atom and a CN group across alkenes **27**. This reaction is applicable not only to a wide range of terminal styrenes but also to internal alkenylarenes, enynes, and allenes, providing enantioenriched products in good yields with high enantioselectivities.

This reaction involves Co-catalyzed HAT and Cu-catalyzed enantioselective radical cyanation. In the proposed catalytic cycle, Co(III)–H species **92** are initially formed from the anodically oxidized Co(III) complex **91** and hydrosilane **88** (Figure 15). Subsequently, the HAT between the Co(III)–H catalyst **92** and the alkene **27** generates a carbon-centered radical species **93** with a newly formed C–H bond. These radical species then enter the second catalytic cycle, facilitating the asymmetric cyanide transfer. The radical species **93** undergo a single oxidative addition to the Cu(II)–CN catalyst **96**, forming a Cu(III) complex. Finally, reductive elimination delivers the enantioenriched nitrile products **89** and a reduced Cu(I) complex **94**, which is reoxidized through anodic oxidation.

**Figure 16:**
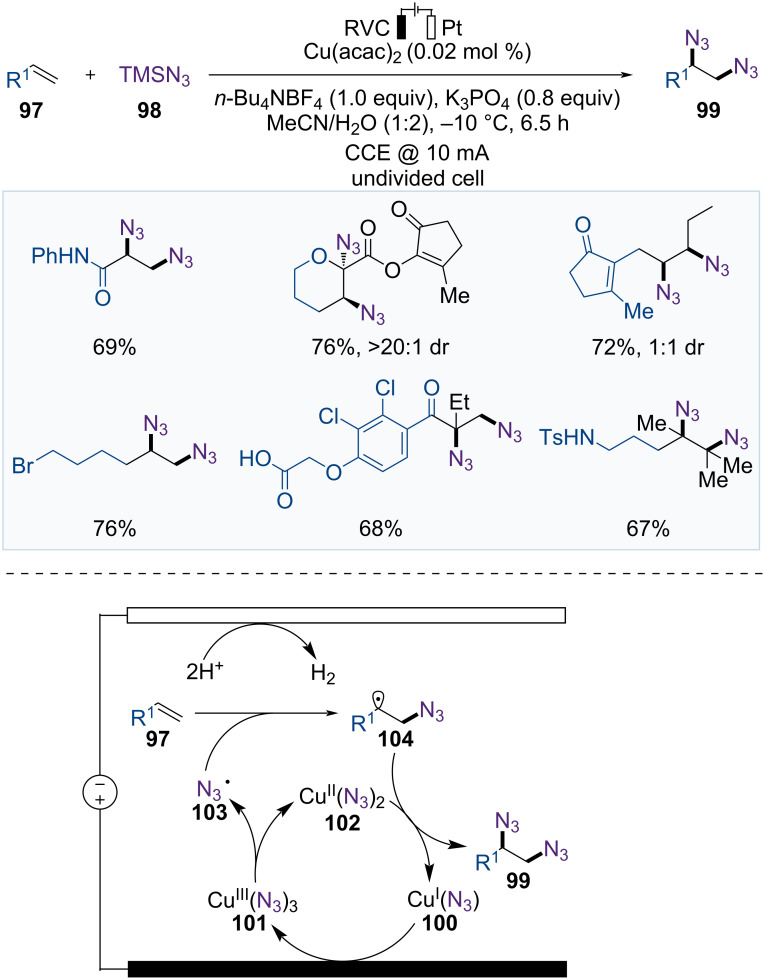
Scheme and proposed mechanism for Cu-catalyzed electrochemical diazidation of olefins.

The 1,2-diamine moiety is present in numerous natural products and bioactive compounds. In 2022, Xu et al. reported the Cu-catalyzed electrocatalytic diazidation of olefins with ppm-level catalyst loading, providing an alternative strategy for 1,2-diamine synthesis (Figure 16) [71]. This reaction successfully expanded the substrate scope from electron-rich to electron-deficient alkenes, which were considered challenging substrates in previous diazidation reactions.

A possible mechanism is proposed in Figure 16. Cu(II)(N_3_)_2_ (**102**) is generated from TMSN_3_ (**98**) and Cu(acac)_2_ in the presence of K_3_PO_4_; this is followed by anodic oxidation to form a Cu(III)(N_3_)_3_ complex **101**. The resulting Cu(III)(N_3_)_3_ complex **101** releases an azide radical (**103**), and Cu(II)(N_3_)_2_ (**102**). The azide radical (**103**) then reacts with the alkene **97** to produce an alkyl radical **104**, which undergoes ligand transfer from Cu(II)(N_3_)_2_ (**102**) to yield the diazidation product **99** and Cu(I)(N_3_) (**100**). The Cu(I)(N_3_) (**100**) is reoxidized to Cu(III)(N_3_)_3_ (**101**) on the anode in the presence of N_3_^-^ to complete the catalytic cycle.

**Figure 17:**
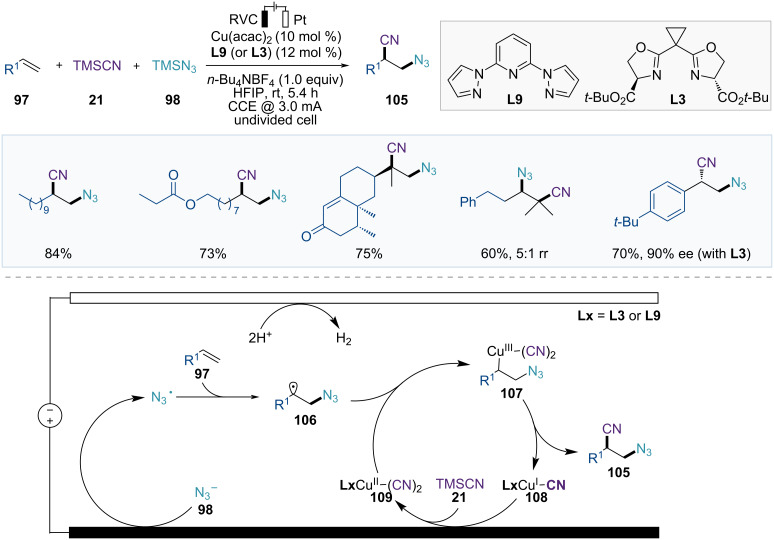
Scheme and proposed mechanism for Cu-catalyzed electrochemical azidocyanation of alkenes.

In 2024, the Xu group developed a Cu-catalyzed electrochemical azidocyanation of alkenes (Figure 17) [72]. This alkene difunctionalization, using TMSCN (**21**) and TMSN_3_ (**98**) as starting materials, features oxidant-free conditions, compatibility with both aryl- and alkylalkenes, and a wide functional group tolerance. Moreover, asymmetric transformations are possible when arylalkenes are used as starting materials in the presence of copper and chiral ligand **L3**, yielding the corresponding chiral products.

Based on mechanistic studies, the catalytic cycle begins with anodic oxidation of N_3_^−^ to generate an azide radical, which adds to the alkene **97** to form carbon-centered radical intermediate **106** (Figure 17). The resulting alkyl radical intermediate **106** then reacts with the Cu(II)(CN)_2_ catalyst **109** to produce a Cu(III) species **107**, which undergoes reductive elimination to deliver the desired product **105** and the Cu(I)CN catalyst **108**. The Cu(II)CN catalyst **109** is regenerated via anodic oxidation to complete its catalytic cycle.

### Decarboxylative functionalization

**Figure 18:**
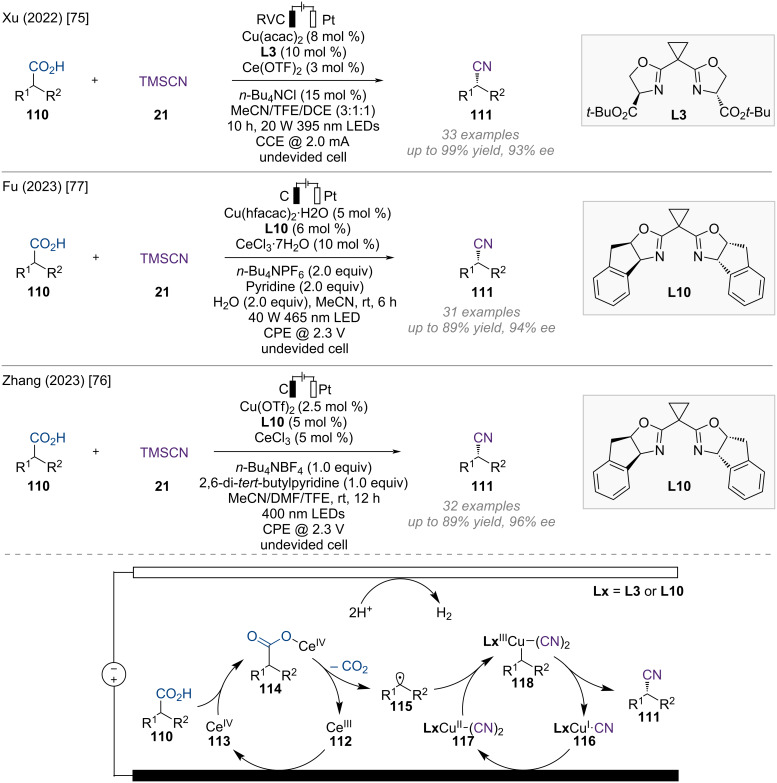
Scheme and proposed mechanism for Cu-catalyzed electrophotochemical asymmetric decarboxylative cyanation.

Carboxylic acids are inexpensive, readily available, structurally diverse from both natural and synthetic sources, and easy to handle. Recently, various catalytic transformations of carboxylic acids have been developed, enabling chemists to access a variety of valuable products via diverse reaction pathways [73]. Particularly, decarboxylative cross-coupling of carboxylic acids using radical strategies has emerged as a robust method for the construction of C–C and C–X bonds [74]. Recently, photoelectrochemical asymmetric decarboxylative cyanation was independently established by the groups of Xu, Zhang, and Fu (Figure 18) [75–77]. Each group employed the same Ce/Cu relay catalysis strategy to produce chiral nitrile compounds in high yields and enantioselectivities. Notably, this method does not require prefunctionalization of carboxylic acids or stoichiometric chemical oxidants.

According to their research, Ce(III) salt **112**, which serves as a photocatalyst, is oxidized to a Ce(IV) complex **113** on the anode (Figure 18). The resulting Ce(IV) species **113** coordinates with carboxylic acid and undergoes photoinduced ligand-to-metal charge transfer (LMCT) and regeneration of the Ce(III) species to produce a benzylic radical **115**. The chiral Cu(II) catalyst **117** reacts with benzylic radical **115** to yield Cu(III) intermediate **118**, which then undergoes reductive elimination to provide the desired enantioenriched nitrile product **111** and Cu(I) catalyst **116**. The resulting Cu(I) catalyst **116** is reoxidized to Cu(II) **117** at the anode, completing the catalytic cycle.

### Coupling reaction (Chan–Lam coupling)

Transition metal-catalyzed C–N bond formation reactions are essential synthetic methodologies. The discovery of Chan–Lam coupling reactions, which use arylboronic acids and *N*-nucleophiles, provided a C–N bond-forming protocol using copper catalysis, offering a complementary method to noble transition-metal catalysis [78]. Recently, dual-catalytic systems combining copper catalysis with electrocatalysis have been developed to avoid the use of chemical oxidants. Thus, the substrate scope was expanded to include electron-deficient arylboronic acids.

**Figure 19:**
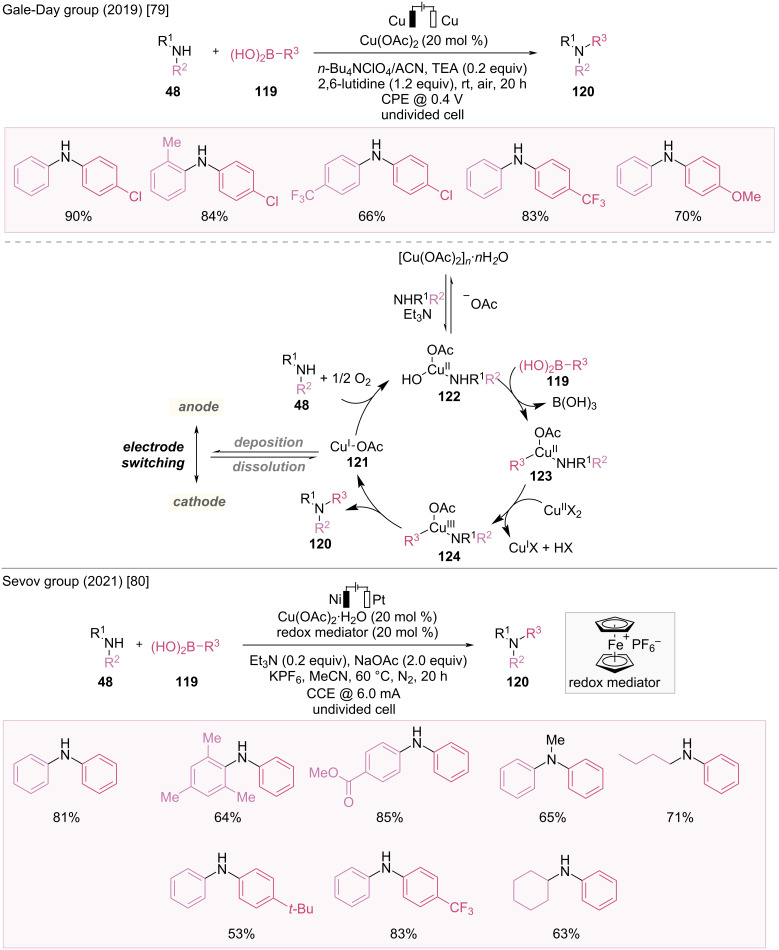
Scheme and proposed mechanism for electrocatalytic Chan–Lam coupling.

In 2019, Gale-Day et al. developed electrocatalytic Chan–Lam couplings of arylboronic acids with primary anilines using a copper catalyst and dual Cu-electrode system to form C–N bonds (Figure 19) [79]. This catalytic system demonstrates a broad substrate scope, including electron-deficient boronic acids, which are typically regarded as challenging substrates because of their low reactivity in Chan–Lam coupling.

The reaction mechanism is illustrated in Figure 19. During the reaction, the unstable Cu(I) species **121** is reduced to Cu(0) at the cathode and is plated to suppress side reactions. After 10 minutes, when the current is inverted, the copper that previously precipitated as an impure film is reoxidized to Cu(I) **121** at the new anode. The Cu(I) species **121** is either oxidized to the Cu(II) complex by oxygen or plated again on the cathode. The Cu(II) catalyst reacts with aniline to produce a Cu(II) intermediate **122**, which then generates a Cu(III) complex **124** through transmetalation of boronic acid and disproportionation. Subsequently, the coupling product **120** is released through reductive elimination, and the resulting Cu(I) species **121** is either reoxidized or plated on the cathode.

Despite the success of electrochemical Chan–Lam coupling for C–N bond formation, the electrooxidative reactions of copper catalysts without ligands face limitations owing to slow electron transfer kinetics, irreversible copper plating, and competing substrate oxidation. To overcome these challenges, Sevov et al. developed a ligand-free, Cu-catalyzed electrochemical Chan–Lam coupling using a ferrocenium salt as a redox mediator with non-copper electrodes (Figure 19) [80]. In the absence of chemical oxidants, this Chan–Lam coupling method resulted in shorter reaction times than those of traditional methods and offered alternative substrate reactivity. This reaction was successfully applied to aryl- and alkylamines with arylboronic acids, achieving high yields. Mechanistic studies revealed that the mediator plays multiple roles, including rapidly oxidizing low-valent Cu intermediates to maintain high Cu(II) concentrations, removing Cu metal from the cathode to regenerate the active Cu catalyst, revealing the Pt surface for proton reduction, and offering anodic overcharge protection to avoid undesirable substrate oxidation.

## Conclusion

Over the past two decades, significant progress has been made in electrochemical organic reactions, supported by the development of more sustainable and versatile methodologies for carbon–carbon and carbon–heteroatom bond formation. These advances have been accomplished by recognizing electrochemistry as an effective and sustainable approach for electron transfer processes to enable the generation of highly reactive intermediates. Additionally, the rapid growth of dual catalytic systems (merging electrochemistry with transition-metal catalysis) has led to the development of new, efficient, and chemo- and stereoselective synthetic transformations.

This review highlighted the recent developments in dual catalytic reactions involving copper and electrocatalysis, including C–H activation, olefin addition, decarboxylative coupling, and Chan–Lam coupling. Although remarkable achievements have been made in this field, significant gaps that require attention remain, and further investigation into new and efficient transformations is necessary. The substrate scope is often limited, which can restrict both reactivity and selectivity. Significant progress has been achieved in C–H activation reactions, with the use of directing groups, which play a crucial role in selectivity and reactivity. However, the use of directing groups requires additional synthetic steps for their installation and removal and present challenges with limited substrate compatibility. Future research could focus on designing catalytic systems that reduce dependence on directing groups, such as utilizing transient directing groups to simplify the synthetic process. Moreover, the development of asymmetric transformations remains challenging due to the difficulty in controlling the stereo- and chemoselectivity of highly reactive radical intermediates. Future efforts should focus on designing new ligand frameworks to broaden substrate scopes and enhance selectivity control. The use of greener solvents is also important for more sustainable reactions. Furthermore, the application of electrochemical copper catalysis to complex molecules, such as for the late-stage functionalization of pharmaceuticals or materials and bioorthogonal functionalization of biomacromolecules, is necessary. Further advances in Cu-catalyzed electrochemical reactions are expected in the near future, developing powerful tools for sustainable and efficient organic syntheses.

## Data Availability

Data sharing is not applicable as no new data was generated or analyzed in this study.

## References

[1] Hassan J, Sévignon M, Gozzi C, Schulz E, Lemaire M (2002). Chem Rev.

[2] Altman R A, Buchwald S L (2007). Nat Protoc.

[3] Ullmann F, Bielecki J (1901). Ber Dtsch Chem Ges.

[4] Ullmann F (1903). Ber Dtsch Chem Ges.

[5] Ullmann F, Sponagel P (1905). Ber Dtsch Chem Ges.

[6] Goldberg I (1906). Ber Dtsch Chem Ges.

[7] Rosenmund K W, Struck E (1919). Ber Dtsch Chem Ges.

[8] Robert W, Hurtley H (1929). J Chem Soc.

[9] Chodkiewicz W (1957). Ann Chim (Cachan, Fr).

[10] Beletskaya I P, Cheprakov A V (2004). Coord Chem Rev.

[11] Biffis A, Centomo P, Del Zotto A, Zecca M (2018). Chem Rev.

[12] Ariafard A, Lin Z (2006). Organometallics.

[13] Kaga A, Chiba S (2017). ACS Catal.

[14] Iqbal J, Bhatia B, Nayyar N K (1994). Chem Rev.

[15] Julia M (1971). Acc Chem Res.

[16] Rathke M W, Lindert A (1971). J Am Chem Soc.

[17] Kozlowski M C, DiVirgilio E S, Malolanarasimhan K, Mulrooney C A (2005). Tetrahedron: Asymmetry.

[18] Kaneda K, Itoh T, Kii N, Jitsukawa K, Teranishi S (1982). J Mol Catal.

[19] Tokunaga M, Shirogane Y, Aoyama H, Obora Y, Tsuji Y (2005). J Organomet Chem.

[20] Li Z-L, Fang G-C, Gu Q-S, Liu X-Y (2020). Chem Soc Rev.

[21] Hossain A, Bhattacharyya A, Reiser O (2019). Science.

[22] Bissember A C, Lundgren R J, Creutz S E, Peters J C, Fu G C (2013). Angew Chem, Int Ed.

[23] Do H-Q, Bachman S, Bissember A C, Peters J C, Fu G C (2014). J Am Chem Soc.

[24] Matier C D, Schwaben J, Peters J C, Fu G C (2017). J Am Chem Soc.

[25] Zhou H, Li Z-L, Gu Q-S, Liu X-Y (2021). ACS Catal.

[26] Zhang Z, Chen P, Liu G (2022). Chem Soc Rev.

[27] Gu Q-S, Li Z-L, Liu X-Y (2020). Acc Chem Res.

[28] Zhang W, Wang F, McCann S D, Wang D, Chen P, Stahl S S, Liu G (2016). Science.

[29] Kainz Q M, Matier C D, Bartoszewicz A, Zultanski S L, Peters J C, Fu G C (2016). Science.

[30] Chen C, Peters J C, Fu G C (2021). Nature.

[31] Liu J, Lu L, Wood D, Lin S (2020). ACS Cent Sci.

[32] Yan M, Kawamata Y, Baran P S (2017). Chem Rev.

[33] Novaes L F T, Liu J, Shen Y, Lu L, Meinhardt J M, Lin S (2021). Chem Soc Rev.

[34] Ackermann L (2020). Acc Chem Res.

[35] Jiao K-J, Xing Y-K, Yang Q-L, Qiu H, Mei T-S (2020). Acc Chem Res.

[36] Wang F, Stahl S S (2020). Acc Chem Res.

[37] Chang X, Zhang Q, Guo C (2020). Angew Chem, Int Ed.

[38] Malapit C A, Prater M B, Cabrera-Pardo J R, Li M, Pham T D, McFadden T P, Blank S, Minteer S D (2022). Chem Rev.

[39] Gandeepan P, Müller T, Zell D, Cera G, Warratz S, Ackermann L (2019). Chem Rev.

[40] Docherty J H, Lister T M, Mcarthur G, Findlay M T, Domingo-Legarda P, Kenyon J, Choudhary S, Larrosa I (2023). Chem Rev.

[41] Wang W, Lorion M M, Shah J, Kapdi A R, Ackermann L (2018). Angew Chem, Int Ed.

[42] Guillemard L, Kaplaneris N, Ackermann L, Johansson M J (2021). Nat Rev Chem.

[43] Son J (2021). Beilstein J Org Chem.

[44] Zhang L, Ritter T (2022). J Am Chem Soc.

[45] Wang Y, Dana S, Long H, Xu Y, Li Y, Kaplaneris N, Ackermann L (2023). Chem Rev.

[46] Bellotti P, Huang H-M, Faber T, Glorius F (2023). Chem Rev.

[47] Sauermann N, Meyer T H, Qiu Y, Ackermann L (2018). ACS Catal.

[48] Tian C, Dhawa U, Scheremetjew A, Ackermann L (2019). ACS Catal.

[49] Zhang Z-Z, Zhou G, Yue Q, Yao Q-J, Shi B-F (2024). ACS Catal.

[50] Gao P-S, Weng X-J, Wang Z-H, Zheng C, Sun B, Chen Z-H, You S-L, Mei T-S (2020). Angew Chem, Int Ed.

[51] Guo B, Xu H-C (2021). Beilstein J Org Chem.

[52] He Z, Liu H-L, Wang Z-H, Jiao K-J, Li Z-M, Li Z-J, Fang P, Mei T-S (2023). J Org Chem.

[53] Lu Q, Glorius F (2017). Angew Chem, Int Ed.

[54] Zhang C, Li Z-L, Gu Q-S, Liu X-Y (2021). Nat Commun.

[55] Cai C-Y, Lai X-L, Wang Y, Hu H-H, Song J, Yang Y, Wang C, Xu H-C (2022). Nat Catal.

[56] Fan W, Zhao X, Deng Y, Chen P, Wang F, Liu G (2022). J Am Chem Soc.

[57] Lai X-L, Xu H-C (2023). J Am Chem Soc.

[58] Xie T, Huang J, Li J, Peng L, Song J, Guo C (2023). Nat Commun.

[59] Zhang J, Zhu W, Chen Z, Zhang Q, Guo C (2024). J Am Chem Soc.

[60] Yang Q-L, Wang X-Y, Lu J-Y, Zhang L-P, Fang P, Mei T-S (2018). J Am Chem Soc.

[61] Kathiravan S, Suriyanarayanan S, Nicholls I A (2019). Org Lett.

[62] Baidya M, De Sarkar S (2023). Org Lett.

[63] Tsuchida K, Kochi T, Kakiuchi F (2013). Asian J Org Chem.

[64] Yang X, Yang Q-L, Wang X-Y, Xu H-H, Mei T-S, Huang Y, Fang P (2020). J Org Chem.

[65] Chen J, Lu Z (2018). Org Chem Front.

[66] Dhungana R K, KC S, Basnet P, Giri R (2018). Chem Rec.

[67] Pitzer L, Schwarz J L, Glorius F (2019). Chem Sci.

[68] Sauer G S, Lin S (2018). ACS Catal.

[69] Fu N, Song L, Liu J, Shen Y, Siu J C, Lin S (2019). J Am Chem Soc.

[70] Song L, Fu N, Ernst B G, Lee W H, Frederick M O, DiStasio R A, Lin S (2020). Nat Chem.

[71] Cai C-Y, Zheng Y-T, Li J-F, Xu H-C (2022). J Am Chem Soc.

[72] Zheng Y-T, Xu H-C (2024). Angew Chem, Int Ed.

[73] Rodríguez N, Goossen L J (2011). Chem Soc Rev.

[74] Laudadio G, Palkowitz M D, El-Hayek Ewing T, Baran P S (2022). ACS Med Chem Lett.

[75] Lai X-L, Chen M, Wang Y, Song J, Xu H-C (2022). J Am Chem Soc.

[76] Yuan Y, Yang J, Zhang J (2023). Chem Sci.

[77] Yang K, Wang Y, Luo S, Fu N (2023). Chem – Eur J.

[78] West M J, Fyfe J W B, Vantourout J C, Watson A J B (2019). Chem Rev.

[79] Wexler R P, Nuhant P, Senter T J, Gale-Day Z J (2019). Org Lett.

[80] Walker B R, Manabe S, Brusoe A T, Sevov C S (2021). J Am Chem Soc.

